# The deubiquitylase Ataxin-3 restricts PTEN transcription in lung cancer cells

**DOI:** 10.1038/onc.2013.512

**Published:** 2013-12-02

**Authors:** J J Sacco, T Y Yau, S Darling, V Patel, H Liu, S Urbé, M J Clague, J M Coulson

**Affiliations:** 1 Cellular and Molecular Physiology, Institute of Translational Medicine, University of Liverpool, Liverpool, UK

**Keywords:** deubiquitinase, ATXN3, phosphatase and tensin homolog, PTENP1, ceRNA, MJD

## Abstract

The phosphatidylinositol-3-kinase (PI3K) pathway is commonly hyperactivated in cancer. One mechanism by which this occurs is by silencing of the phosphatase and tensin homolog (PTEN), a tumor suppressor and major antagonist of the pathway, through genetic, epigenetic or posttranscriptional mechanisms. Here, we used an unbiased siRNA screen in non-small-cell lung cancer cells to identify deubiquitylases (DUBs) that have an impact on PI3K signaling by regulating the abundance of PTEN. We found that PTEN expression was induced by depleting any of three members of the Josephin family DUBs: ataxin 3 (ATXN3), ataxin 3-like (ATXN3L) and Josephin domain containing 1 (JOSD1). However, this effect is not mediated through altered PTEN protein stability. Instead, depletion of each DUB increases expression of both the PTEN transcript and its competing endogenous RNA, PTENP1. In ATXN3-depleted cells, under conditions of transcriptional inhibition, PTEN and PTENP1 mRNAs rapidly decay, suggesting that ATXN3 acts primarily by repressing their transcription. Importantly, the PTEN induction observed in response to ATXN3 siRNA is sufficient to downregulate Akt phosphorylation and hence PI3K signaling. Histone deacetylase inhibitors (HDACi) have been suggested as potential mediators of PTEN transcriptional reactivation in non-small-cell lung cancer. Although PTEN exhibits a very limited response to the broad-spectrum HDACi Vorinostat (SAHA) in A549 cells, we find that combination with ATXN3 depletion enhances PTEN induction in an additive manner. Similarly, these interventions additively decrease cell viability. Thus, ATXN3 provides an autonomous, complementary therapeutic target in cancers with epigenetic downregulation of PTEN.

## Introduction

The phosphatidylinositol-3-kinase (PI3K) pathway is frequently dysregulated in cancer and is a prime target for oncology drug development. Signaling is initiated by the binding of ligand to membrane receptors, leading to the activation of PI3-kinases, and the generation of the lipid second messenger PtdIns(3,4,5)*P*_3._ Activation of this pathway is finely balanced by the tumor suppressor phosphatase and tensin homolog (PTEN),^1^ which catalyzes the dephosphorylation of PtdIns(3,4,5)*P*_3_, leading to inactivation of the pathway.

PTEN is one of the most frequently silenced tumor suppressors^2, 3^ and is haploinsufficient,^4, 5^ with subtle changes in PTEN expression altering cancer susceptibility.^6^ PTEN is disrupted in many sporadic tumors as well as in cancer predisposition syndromes,^7, 8^ and PTEN knockout mice show increased susceptibility to multiple malignancies.^9, 10, 11^ PTEN exhibits both lipid and protein phosphatase activity and has manifold tumor suppressor functions (reviewed in Song and Salmena^3^), including a major role in dephosphorylation of PtdIns(3,4,5)*P*_3_.^1^ Loss of PTEN increases Akt phosphorylation and deregulates PI3K signaling, which in turn increases cell survival.^12, 13^ Silencing of PTEN occurs not only by genetic mutation but also through alternative mechanisms, including regulation at transcriptional and posttranscriptional levels (reviewed in Leslie and Foti^14^). Indeed, PTEN is the paradigm for a new mechanism of posttranscriptional regulation exerted by competitive endogenous RNAs (ceRNAs), which are co-regulated with other transcripts bearing common microRNA recognition elements.^15, 16^ In lung adenocarcinoma, although PTEN inactivation by mutation is rare,^17^ loss of PTEN expression is not uncommon and has been attributed to both epigenetic^18^ and posttranscriptional^19^ mechanisms. Thus, reactivation of PTEN, potentially achievable by promoting PTEN expression, stability or enzymatic activity, may provide a valuable therapeutic strategy (reviewed in Leslie and Foti^14^).

Deubiquitylases (DUBs) are a family of approximately 90 enzymes that catalyze the removal of ubiquitin from protein substrates and regulate several aspects of protein fate. Reversible ubiquitylation is involved in regulating diverse cellular processes, including many germane to cancer.^20, 21, 22, 23, 24^ Several components of the PI3K pathway are ubiquitylated, including PTEN, which is both monoubiquitylated and polyubiquitylated, leading to nuclear localization and proteasomal degradation, respectively.^25, 26, 27^ The DUB USP7 can reverse PTEN monoubiquitylation, affecting its cellular localization;^28^ however, to date no DUBs have been described that reverse PTEN polyubiquitylation or otherwise influence PTEN expression levels.

To identify specific DUBs involved in regulating the cellular level of PTEN protein, we performed an unbiased siRNA screen that systematically assessed the effect of depleting each DUB. Three closely related Josephin sub-family DUBs were identified, including ATXN3, each of whose depletion increased PTEN transcription. Depletion of ATXN3 was sufficient to attenuate Akt phosphorylation and enhance the ability of histone deacetylase inhibitors (HDACi) to induce PTEN expression and decrease cell viability, suggesting ATXN3 inhibition may be of therapeutic benefit in cancers with nongenetic inactivation of PTEN.

## Results and Discussion

**Figure 1:**
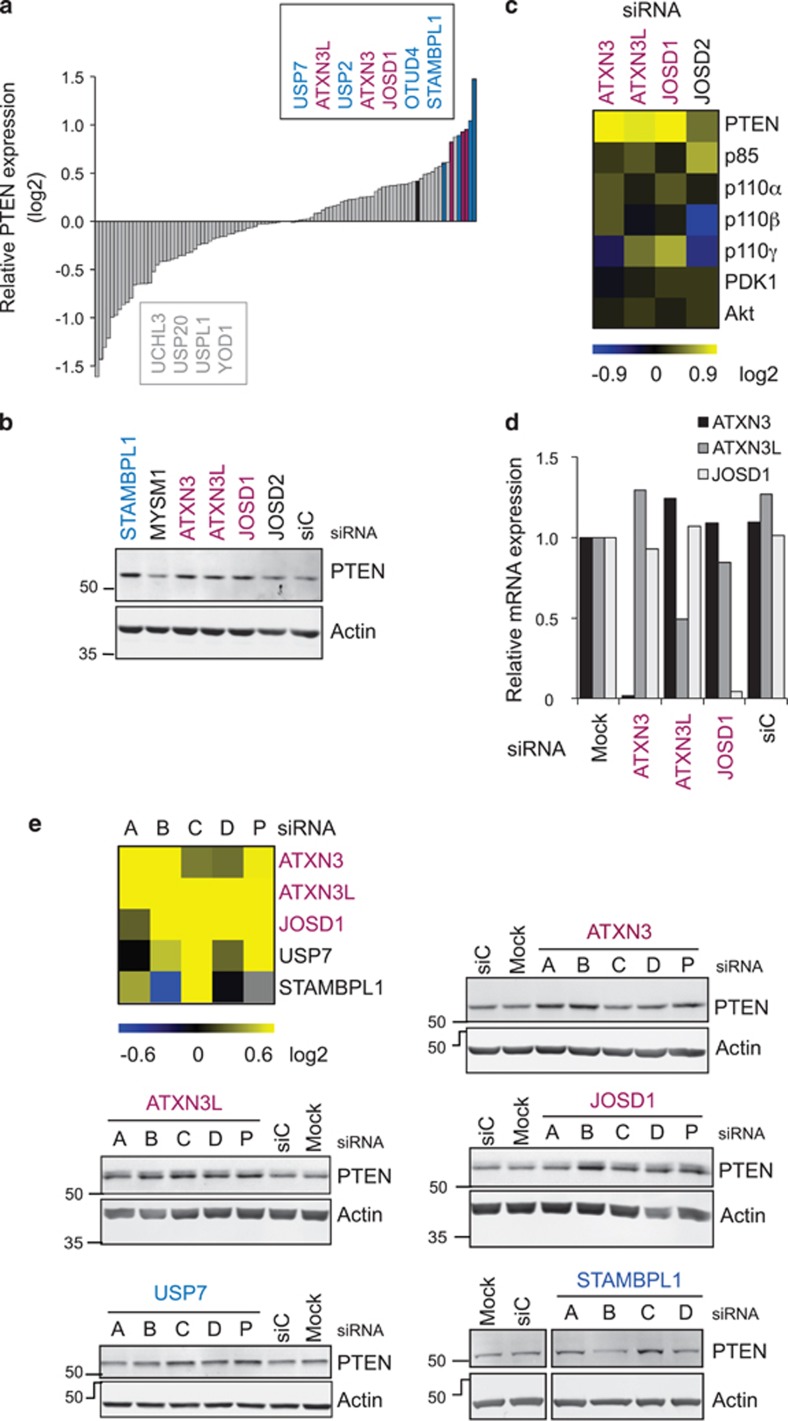
A siRNA screen identifies Josephin family DUBs as negative regulators of PTEN abundance. (**a** and **b**), Identification of ATXN3, ATXN3L and JOSD1 from an unbiased DUB siRNA screen. A549 cells (from ECACC and cultured in DMEM with 10% FBS, 1% NEAA, 100 U/ml penicillin/streptomycin at 37 °C and 5% CO_2_) were seeded at 9 × 10^7^ cells in 150 mm dishes and transfected with a library of pooled siRNA oligos targeting 92 human DUBs (Qiagen), or AllStars non targeting control siRNA (Qiagen, siC), or no siRNA (Mock), at a final concentration of 40 nM using Oligofectamine (Invitrogen). Proteins were extracted 72 h later and equal amounts of lysates (determined by BCA assay, Thermo Scientific) were arrayed in 96-well plates. Proteins extracted in NP40 buffer (0.5% NP40, 25 mM Tris pH7.9, 150 mM NaCl with protease inhibitors (Roche)) were resolved on four 8% SDS-PAGE gels, transferred to BiotraceNT membrane (VWR) and immunoblotted with mouse anti-PTEN (Santa Cruz, sc-7974) and mouse anti-β-actin (ab6276, Abcam) antibodies. Proteins were visualized using IRDye-conjugated secondary antibodies and a LI-COR Odyssey 2.1; 16-bit images were quantified using Odyssey analysis software. The PTEN levels were expressed relative to actin and then normalized to the median of each immunoblot, before collation and ranking of the log2 transformed data. (**a**), The entire data set ranked by PTEN abundance, highlighting DUBs selected for deconvolution (blue), including the Josephins ATXN3, ATXN3L and JOSD1 (shown in maroon); JOSD2 is shown in black. (**b**), A portion of an immunoblot from the screen, showing PTEN levels after depletion of the Josephin DUBs or the nontargeting control (siC). (**c**), Josephin DUBs do not coordinately regulate other PI3K pathway components. The entire NP40 lysate library was immunoblotted for p85 (BD Biosciences, 610045), p110α (Cell Signaling, 4249), p110β (Cell Signaling, 3011), p110γ (Cell Signaling, 5405), PDK1 (BD Biosciences, 611071) and total Akt (Cell Signaling, 9272) and expression was quantified as before; log2 transformed data for the Josephin DUBs are represented in a heatmap. (**d**), Josephin family DUB siRNAs are target specific. A549 cells were seeded in six-well plates at 1 × 10^5^ cells per well and the following day transfected with the indicated siRNA pools (10 nM) using Lipofectamine RNAiMAX (Invitrogen). Total RNA was extracted 48 h later using RNeasy columns with on-column DNase digestion (Qiagen). cDNA was reverse transcribed from 1 μg RNA with RevertAid H-minus M-MuLV reverse transcriptase (Fermentas) using an oligodT primer (Promega). qRT-PCR was performed in triplicate using SYBR Green Supermix and a CFX system (Biorad). PCR primer pairs are listed in Supplementary Table 1. Samples underwent two-step amplification at 94 °C (30 s) and 60 °C (60 s); melt curves were analyzed after 40 cycles. The Ct values for test genes were normalized to actin and relative expression represented as 2^−[ΔΔCt]^. (**e**), Deconvolution of siRNA pools supports a role for three Josephin DUBs in the negative regulation of PTEN. A549 cells were seeded in six-well plates at 6 × 10^4^ cells per well and the following day transfected for 72 h with each of the four individual oligos (A-D; 10 nM) that comprised each siRNA pool (P) using Lipofectamine RNAiMAX (Invitrogen). Data are shown for five DUBs whose depletion enhanced PTEN expression in the initial screen. Equal amounts of each lysate were immunoblotted for PTEN and actin. The heatmap summarizes the normalized PTEN expression; scale limits represent 1.5-fold change.

We undertook an unbiased screen to identify members of the DUB superfamily that influence the levels of PTEN expression in a non-small-cell lung cancer cell line. We utilized A549 cells as they retain sufficient wild-type PTEN expression to restrict basal Akt phosphorylation despite a homozygous activating KRAS mutation but exhibit phosphorylation of Akt in response to EGF stimulation.^29^ A DUB siRNA library consisting of pools of four oligonucleotides for each of 92 human DUBs was designed in collaboration with Qiagen (Manchester, UK; library details available on request). Cells were transfected with these siRNA pools or with control reagents and nontargeting siRNAs. Libraries of cellular proteins were prepared 72 h later by sequential extraction of cytosol, nucleoplasm and the residual pellet, which were arrayed into 96-well plates. PTEN was only detectable in the cytosol fraction (data not shown), which was utilized for this siRNA screen. The effect of depleting each DUB on PTEN abundance is shown ranked in Figure 1a.

In view of the potential therapeutic applications for reactivating PTEN, we concentrated on the DUBs whose depletion elevated PTEN levels most markedly. Interestingly, among these were three of the four members of the Josephin sub-family, also known as the Machado Joseph Disease sub-family: ATXN3, ATXN3L and JOSD1 (Figure 1b). Each of these ranked above USP7 (HAUSP), a DUB that was previously implicated in the regulation of PTEN monoubiquitylation and sub-cellular localization.^28^ Of note, the Josephin DUBs did not significantly or coordinately increase the expression of other PI3K pathway components (Figure 1c). We used quantitative reverse-transcription PCR (qRT-PCR) to confirm that each siRNA pool was specific for the intended Josephin family target, and found no evidence for crossreactivity of these siRNAs between the family members (Figure 1d). To assess whether off-target effects might be responsible for altering PTEN abundance, we transfected the four independent sequences that comprised each siRNA pool. Statistically, it is unlikely that more than one siRNA within a pool would have the same off-target effect. Our criteria for deconvolution were thus that at least two individual siRNAs produced a greater than 1.5-fold induction of PTEN. However, for STAMBPL1, only one siRNA sequence replicated the induction seen (Figure 1e) in the initial screen, suggesting that this could be an off-target effect. STAMBPL1 and other DUBs from the screen that failed to deconvolute (data not shown) were not pursued further. In contrast, multiple siRNAs for each of the three Josephin DUBs led to robust induction of PTEN and, overall, 9 out of the 12 siRNAs met our criteria (Figure 1e). As in the original screen, depletion of each Josephin DUB more markedly influenced PTEN levels compared with siRNA-targeting USP7.

**Figure 2:**
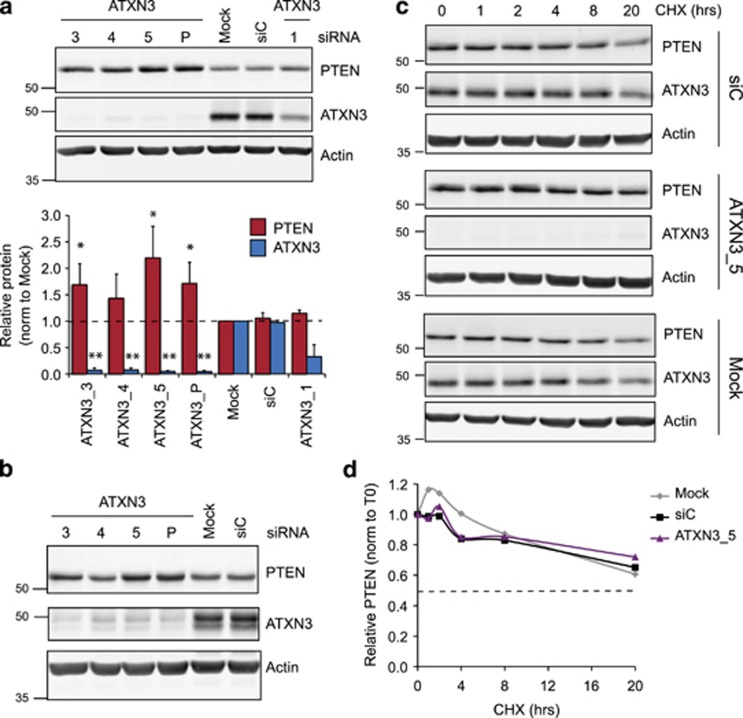
Expression but not stability of PTEN protein is dependent on ATXN3 abundance. (**a**), Efficient ATXN3 depletion is required to induce PTEN expression. A549 cells in six-well plates were transfected for 72 h with new batches of the indicated ATXN3 siRNAs (10 nM, sequences listed in Supplementary Table 2). After 72 h, cells were lysed in NP40 buffer and equal amounts of lysate immunoblotted using mouse anti-ATXN3 (BioLegend, 650401), PTEN and actin antibodies as before; a representative experiment is shown (top). PTEN and ATXN3 were quantified relative to actin and normalized to the mock control (below); mean data are shown from three (ATXN3) or four (PTEN) independent experiments, and error bars show standard deviation. The Student *t*-test compared PTEN or ATXN3 abundance in response to each siRNA with that with siC, *P<0.05, **P<0.001. (**b**), ATXN3 depletion induces PTEN in KRAS wild-type non-small-cell lung cancer. NCI-H322 cells (from CR-UK and cultured in RPMI with 10% FBS) were transfected and analyzed as described above (72 h). (**c** and **d**), ATXN3 depletion does not alter the stability of PTEN in the absence of translation. A549 cells were transfected for a total of 72 h with ATXN3, nontargeting (siC), or no (Mock) siRNA for a minimum of 52 h before treatment for the indicated duration with 100 μg/ml cycloheximide (CHX, Sigma). Protein turnover was monitored over an initial 8 h chase up to a maximum of 20 h; cells were lysed in NP40 buffer and processed for immunoblotting. (**c**), Equal amounts of lysate were immunoblotted for PTEN, ATXN3 or actin. (**d**), PTEN was quantified relative to actin and normalized to the amount in each sample prior to starting the cycloheximide treatment. The half-life, indicated by a dotted line, is >20 h for PTEN.

ATXN3 is the best studied of the Josephin DUBs and is a therapeutic target in neurodegenerative disease.^30^ For these reasons, together with the availability of good antibodies, we focused our attention on characterizing the mechanism by which ATXN3 regulates PTEN. A new batch of siRNAs was employed, three of which (siATXN3_3, _4 and _5) depleted virtually all cellular ATXN3, whereas the fourth (siATXN3_1) was less effective with 40% of ATXN3 remaining. Importantly, the induction of PTEN correlated with the efficacy of ATXN3 depletion (Figure 2a). The marginal effect with 60% depletion suggests that cellular ATXN3 may be in excess of that required to restrict PTEN expression. We also knocked down ATXN3 in a second non-small-cell lung cancer cell line, NCI-H322, which has wild-type KRAS. NCI-H322 cells exhibited a similar response to the KRAS-mutant A549 cells, with around twofold induction of PTEN for the most efficacious ATXN3 siRNAs (Figure 2b). Thus, PTEN levels are dependent on those of ATXN3 and this is independent of KRAS status.

We now sought to understand how ATXN3 regulates the levels of PTEN. One obvious mechanism by which DUBs may affect the cellular abundance of proteins is through antagonizing their polyubiquitylation and degradation by the proteasome. However, in this case, loss of DUBs increases rather than decreases PTEN abundance. Nevertheless, we carried out a cycloheximide chase to investigate whether ATXN3 depletion altered the turnover of PTEN. Estimates of PTEN protein half-life vary widely in different cell lines and conditions, ranging from less than 10 h to more than 72 h.^31, 32, 33, 34, 35^ We found that the half-life of PTEN was greater than 20 h in A549 cells, and the half-life of ATXN3 was of a similar order (Figure 2c). Although ATXN3 depletion elevated PTEN levels (Figure 2c), it did not influence the rate of PTEN turnover (Figure 2d), suggesting that its role is independent of direct PTEN ubiquitylation.

**Figure 3:**
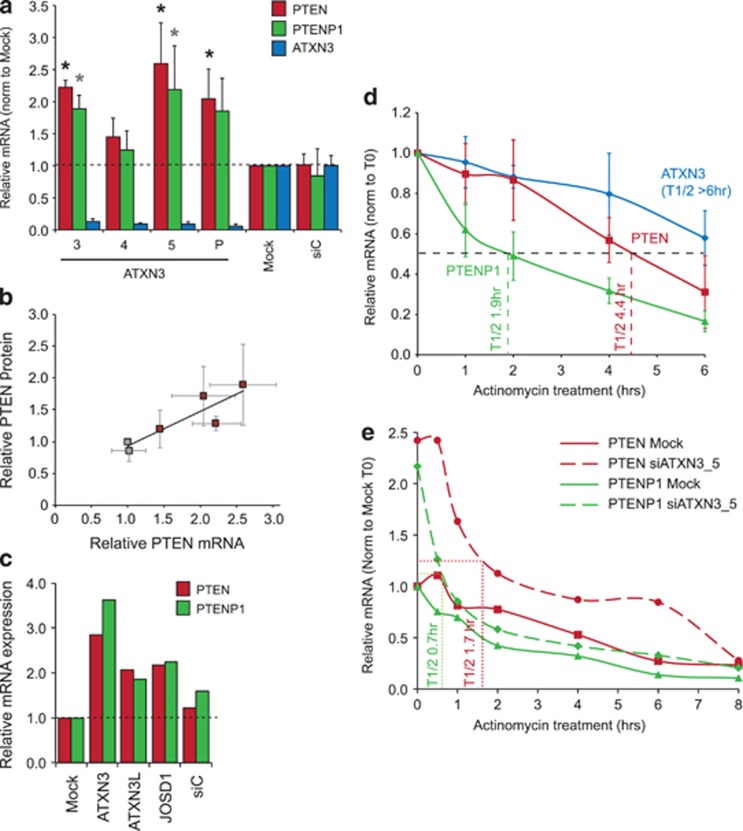
ATXN3 regulates PTEN and PTENP1 transcript abundance. (**a**), ATXN3 depletion markedly induces PTEN and PTENP1 transcript levels. A549 cells were seeded in six-well plates at 1 × 10^5^ cells per well and transfected the following day with the indicated siRNAs (10 nM) for 48 h prior to extraction of total RNA. Expression of ATXN3, PTEN and PTENP1 transcripts (PCR primer pairs are listed in Supplementary Table 1) relative to actin were determined by qRT-PCR. Mean values are shown, normalized to those for mock-transfected cells, from three independent experiments; error bars show standard deviation. The Student *t*-test compares each siRNA with siC, *P<0.05. (**b**), PTEN mRNA is a good predictor of PTEN protein expression. A549 cells were transfected with the indicated siRNAs for 48 h and then lysed in NP40 buffer and analyzed by immunoblotting. PTEN and ATXN3 were quantified relative to actin and normalized to the mock control; the scatter plot correlates PTEN mRNA expression (shown in panel **a**) with PTEN protein expression (*n*=3, error bars show standard deviation, R^2^=0.797); controls are shown in grey and ATXN3 knockdown samples in maroon. (**c**), Josephin family DUBs independently induce PTEN mRNA expression. Total RNA from siRNA-transfected A549 cells (see experiment in Figure 1d) was subject to qRT-PCR for PTEN and PTENP1. (**d**), Degradation of the PTEN transcript is delayed relative to that of the PTENP1 transcript in the absence of transcription. A549 cells were treated with 5 μg/ml actinomycin D (Sigma) for the indicated times prior to preparation of total RNA. Transcripts were quantified relative to actin by qRT-PCR. Mean values of three independent experiments, normalized to the expression level of each gene prior to addition of actinomycin D, are shown. Error bars show standard deviation; the half-life is indicated by a dotted line. (**e**), Depletion of ATXN3 increases the levels but decreases the stability of PTEN and PTENP1 transcripts. A549 cells were transfected with siRNA for a total of 48 h; cells were treated with 5 μg/ml actinomycin D for the indicated times immediately prior to preparation of total RNA. Transcript expression was determined by qRT-PCR over an 8 h chase; mean values are shown from two independent experiments. The half-life of each transcript following ATXN3 depletion is indicated by a fine dotted line.

Many DUBs have been shown to act at the level of RNA processing^36, 37^ and ATXN3 has an established role as a transcriptional repressor.^38, 39^ We next investigated whether ATXN3 might influence the levels of the PTEN transcript. As some reduction in cell numbers was apparent after 72 h, transcripts were analyzed after 48 h ATXN3 depletion. We found that PTEN mRNA was significantly increased compared with control cells (Figure 3a). The effect of ATXN3 depletion on PTEN protein level was also apparent after 48 h, and strongly correlated with the PTEN RNA level in parallel samples (Figure 3b). Thus, ATXN3 depletion leads to increased PTEN transcript, which in turn leads to elevated cellular PTEN protein. However, the regulation of PTEN mRNA is highly complex, in part due to co-regulation with ceRNAs.^15, 16^ Networks of ceRNAs act as microRNA sponges, so that when a given transcript is overexpressed cellular concentrations of certain microRNA recognition elements are increased and can result in the de-repression of other transcripts containing the same microRNA recognition elements.^40^ The first proven ceRNA, and that most tightly linked to PTEN, is the highly homologous pseudogene PTENP1.^41^ We therefore assessed whether the PTENP1 transcript was also responsive to ATXN3 depletion and found that it closely mirrored the response of the PTEN mRNA (Figure 3a). Therefore, ATXN3 may directly regulate the PTEN transcript, or indirectly affect it through regulation of a ceRNA such as PTENP1.

Interestingly, the three DUBs that were validated by deconvolution from the initial screen (Figure 1) are all members of the Josephin/Machado Joseph Disease family, which consists of only four cysteine proteases that share a catalytic Josephin domain. We tested whether these three DUBs function towards PTEN in a similar manner, and found that the individual depletion of ATXN3, ATXN3L or JOSD1 in each case induced both PTEN and PTENP1 mRNA levels by twofold or more (Figure 3c). This suggests that, whether by direct or indirect mechanisms, the regulation of PTEN transcription may be a conserved function of this enzyme family.

To establish whether ATXN3 primarily influences the rate of transcription or the rate of transcript degradation, we performed an actinomycin D chase. The PTENP1 transcript was markedly less stable than that for PTEN (Figure 3d), consistent with a role for elevated PTENP1 in sustaining PTEN expression. In ATXN3-depleted cells, the twofold increase in PTEN/PTENP1 transcript levels was not accompanied by an increase in PTEN/PTENP1 mRNA stability; in fact, both transcripts exhibited an increase in their initial turnover and a reduced half-life (Figure 3e). This rapid re-equilibration in the absence of new transcription is consistent with a transcriptional mechanism for ATXN3. Notably in ATXN3-deficient cells, PTENP1 was again least stable, rapidly degrading during the first 30 min of transcriptional arrest, whereas PTEN turnover commenced after 30 min. Despite a pool of PTEN mRNA that exhibits a shorter half-life in ATXN3-depleted cells, a second more stable pool persisted for at least 6 h, perhaps indicating a secondary effect of ATXN3 on PTEN transcript stability.

**Figure 4:**
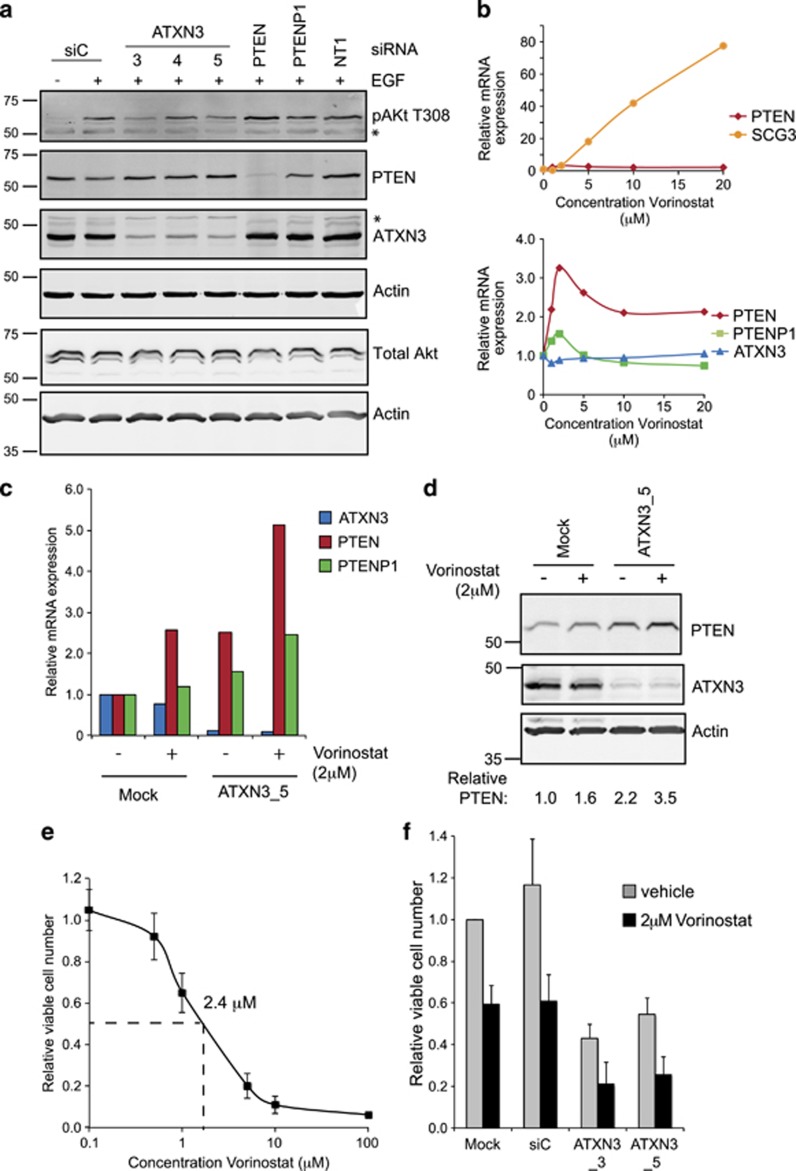
Reactivating PTEN expression and PI3K pathway inhibition through ATXN3 depletion and HDAC inhibition. (**a**), ATXN3 depletion limits Akt phosphorylation. A549 cells were transfected with siRNAs as indicated (NT1, nontargeting control). After 48 h cells were stimulated with 0.5 ng/ml EGF (Preprotech) for 5 min and lysed in ice-cold NP40 buffer (0.5% NP40, 25 mM Tris pH7.9, 100 mM NaCl, 50 mM NaF, with protease and phosphatase inhibitors (Roche)). Samples were processed for immunoblotting with rabbit anti-phospho-Akt (T308, Cell Signaling, 9275) and other antibodies as before. An asterisk indicates nonspecific bands. (**b**), PTEN transcription shows a limited biphasic response to HDAC inhibition. A549 cells were treated with increasing concentrations of Vorinostat (Selleck) for 16 h prior to preparation of total mRNA. Transcript levels were determined by qRT-PCR and are shown normalized to the vehicle control (below). SCG3 is included as an example of a transcript that is highly responsive to HDAC inhibition (above). PCR primer pairs are listed in Supplementary Table 1. (**c** and **d**), ATXN3 depletion enhances the PTEN response to HDAC inhibition. A549 cells were transfected with the indicated siRNAs for 48 h and treated with vehicle or 2 μM Vorinostat for the final 16 h prior to preparation of total mRNA or protein extracts. (**c**), Transcripts were quantified by qRT-PCR and are shown normalized to the level in mock-transfected cells. Bars show the mean values obtained from two independent experiments. (**d**), In a parallel experiment to that shown in panel c, protein was extracted in NP40 buffer and immunoblotted for ATXN3 and PTEN. (**e** and **f**), ATXN3 depletion and HDAC inhibition are additive with respect to cell viability. A549 cells were transfected as indicated with siRNA for 72 h and treated with vehicle (0.04% DMSO) or Vorinostat for the final 48 h prior to analysis. Viable cell numbers were determined based on proportionality with the amount of ATP present, measured using the CellTitre-Glo assay (Promega). Mean values are derived from three independent experiments and normalized to vehicle controls; the error bars show standard deviation. (**e**) In siC-transfected cells the LC_50_ for Vorinostat is 2.4 μM. (**f**), ATXN3 depletion reduces viable cell numbers and works additively with HDAC inhibition. In each case, Vorinostat significantly reduced cell viability relative to the vehicle control, and ATXN3 siRNAs significantly reduced cell viability relative to siC (*P*<0.05).

As PTEN reactivation may have therapeutic applications in cancer,^14^ we next asked whether ATXN3-dependent PTEN induction has an impact on PI3K signaling. To this end, we depleted ATXN3 and monitored Akt phosphorylation in response to acute EGF stimulation. In this experiment, PTEN depletion increased Akt phosphorylation, demonstrating that PTEN is functional and partially restricts PI3K signaling in A549 cells (Figure 4a). In contrast, the two individual ATXN3 siRNAs (siATXN3_3 and _5) that caused the most profound PTEN induction (Figures 2a and 3a) blunted Akt T308 phosphorylation in response to EGF (Figure 4a).

As PTEN expression may be restricted by histone deacetylation, HDACi have been proposed as therapeutic tools to reactivate PTEN.^42, 43^ We investigated the efficacy of this approach in A549 cells using the broad-spectrum HDACi Vorinostat (SAHA), which is licensed for clinical use in cutaneous T-cell lymphoma.^44^ SCG3, a transcript that is tightly repressed by REST^45^ in an HDAC-dependent manner, shows a robust dose-dependent response over a low micromolar range of Vorinostat in A549 cells (Figure 4b). In contrast, PTEN and PTENP1 transcripts exhibit a non-monotonic dose–response curve, with a maximal response at 2 μM of threefold increased PTEN and 1.5-fold increased PTENP1 (Figure 4b). Thus, in isolation, ATXN3 depletion or Vorinostat stimulation can achieve comparable PTEN mRNA induction. Importantly, however, combining the two treatments had an additive effect, resulting in an almost sixfold increase in the PTEN transcript (Figure 4c). At the protein level, ATXN3 siRNA was in fact a more potent inducer of PTEN than Vorinostat and, in combination, an additive 3.5-fold increase in PTEN protein was reached (Figure 4d).

We did not observe cytotoxicity during 16 h Vorinostat treatment. To ascertain the lethal concentration, we extended the period of drug exposure to 48 h; the effective LC_50_ for Vorinostat in A549 cells was 2.4 μM (Figure 4e), similar to the optimal concentration for PTEN induction (Figure 4b). We therefore assessed the phenotypic effects of ATXN3 depletion on cell viability by depleting ATXN3 for 72 h, either alone or in conjunction with 48 h in 2 μM Vorinostat. Either treatment reduced the viable cell number by approximately 50%. However, combining ATXN3 depletion with HDAC inhibition decreased the number of residual viable cells to less than 25% (Figure 4f), in line with the additive effects we observed for induction of PTEN expression (Figures 4c and 4d).

Through unbiased siRNA screening we have identified, for the first time, DUBs that regulate the expression of PTEN. These closely related Josephin DUBs restrict PTEN transcription. ATXN3 is by far the best characterized of the three, although JOSD1 was recently shown to be membrane-associated and to influence both cellular motility and endocytosis.^46^ To date, no specific function has been ascribed to ATXN3L, although its depletion decreases cell motility^47^ and it is reportedly a more efficient enzyme compared with ATXN3.^48^ ATXN3 is a bona fide DUB with a preference for K63-linked ubiquitin chains,^49^ whose catalytic activity is regulated by ubiquitylation.^50^ Interestingly, ATXN3 has been reported to repress transcription through recruitment of HDAC3 to target promoters^38^ or by inhibiting a histone acetyltransferase.^39^ The additive effect of ATXN3 depletion and HDACi on PTEN expression suggest that ATXN3 may regulate PTEN through an HDAC-independent transcriptional or posttranscriptional mechanism.

It is intriguing that three Josephin DUBs can each restrict PTEN transcription. JOSD3 (TAFD3/SL1) is a distantly related Josephin domain containing protein that is not an active DUB^23^ but acts as a global transcriptional activator through association with RNA polymerase-I and its promoter recognition complex.^37, 51^ It is tempting to speculate that certain transcriptional machinery interactions may be conserved in other Josephin DUBs and could mediate their specific repressor activity towards PTEN and/or PTENP1. In fact, ATXN3 interacts with and inhibits CREB-binding protein,^39^ a histone acetyl transferase implicated in PTEN promoter activation.^52^ Cellular concentrations of CREB-binding protein are limiting, so that sequestration of CREB-binding protein could prevent its recruitment to specific promoters, as previously described for suppression of PTEN expression by p65/RelA.^52^ Interestingly, in the context of interplay between ATXN3 depletion and HDAC inhibition, somatic mutations of CREB-binding protein sensitize diffuse large B-cell lymphoma to Vorinostat.^53^

Importantly, as PTEN is frequently silenced in cancer through non genomic mechanisms and even small changes in PTEN expression affect outcome, its pharmaceutical reactivation is likely to prove a valuable therapeutic strategy. Targeting the Josephin DUBs provides a novel mechanism by which this may be achieved. ATXN3 is a key therapeutic target in neurodegeneration, as poly-glutamine expansion of ATXN3 causes Machado-Joseph Disease.^54^ Strategies to target ATXN3 include the use of *in vivo* RNA interference, which can effectively knockdown ATXN3 in mice,^30^ and a Rho-kinase inhibitor reported to promote degradation of poly-glutamine-expanded ATXN3.^55^ Combination therapy, with ATXN3 inhibitors and HDACi, is thus a realistic proposition for PTEN-repressed cancers including lung adenocarcinoma.

## References

[bib1] Maehama T Dixon JE The tumor suppressor, PTEN/MMAC1, dephosphorylates the lipid second messenger, phosphatidylinositol 3,4,5-trisphosphate J Biol Chem 1998 273 13375 13378 9593664 10.1074/jbc.273.22.13375

[bib2] Salmena L Carracedo A Pandolfi PP Tenets of PTEN tumor suppression Cell 2008 133 403 414 18455982 10.1016/j.cell.2008.04.013

[bib3] Song MS Salmena L Pandolfi PP The functions and regulation of the PTEN tumor suppressor Nat Rev Mol Cell Biol 2012 13 283 296 22473468 10.1038/nrm3330

[bib4] Di Cristofano A De Acetis M Koff A Cordon-Cardo C Pandolfi PP Pten and p27KIP1 cooperate in prostate cancer tumor suppression in the mouse Nat Genet 2001 27 222 224 11175795 10.1038/84879

[bib5] Di Cristofano A Kotsi P Peng YF Cordon-Cardo C Elkon KB Pandolfi PP Impaired Fas response and autoimmunity in Pten^+/−^ mice Science 1999 285 2122 2125 10497129 10.1126/science.285.5436.2122

[bib6] Alimonti A Carracedo A Clohessy JG Trotman LC Nardella C Egia A Subtle variations in Pten dose determine cancer susceptibility Nat Genet 2010 42 454 458 20400965 10.1038/ng.556 PMC3118559

[bib7] Li J Yen C Liaw D Podsypanina K Bose S Wang SI PTEN, a putative protein tyrosine phosphatase gene mutated in human brain, breast and prostate cancer Science 1997 275 1943 1947 9072974 10.1126/science.275.5308.1943

[bib8] Steck PA Pershouse MA Jasser SA Yung WK Lin H Ligon AH Identification of a candidate tumor suppressor gene, MMAC1, at chromosome 10q23.3 that is mutated in multiple advanced cancers Nat Genet 1997 15 356 362 9090379 10.1038/ng0497-356

[bib9] Di Cristofano A Pesce B Cordon-Cardo C Pandolfi PP Pten is essential for embryonic development and tumor suppression Nat Genet 1998 19 348 355 9697695 10.1038/1235

[bib10] Suzuki A de la Pompa JL Stambolic V Elia AJ Sasaki T del Barco Barrantes I High cancer susceptibility and embryonic lethality associated with mutation of the PTEN tumor suppressor gene in mice Curr biol 1998 8 1169 1178 9799734 10.1016/s0960-9822(07)00488-5

[bib11] Podsypanina K Ellenson LH Nemes A Gu J Tamura M Yamada KM Mutation of Pten/Mmac1 in mice causes neoplasia in multiple organ systems Proc Natl Acad Sci USA 1999 96 1563 1568 9990064 10.1073/pnas.96.4.1563 PMC15517

[bib12] Stambolic V Suzuki A de la Pompa JL Brothers GM Mirtsos C Sasaki T Negative regulation of PKB/Akt-dependent cell survival by the tumor suppressor PTEN Cell 1998 95 29 39 9778245 10.1016/s0092-8674(00)81780-8

[bib13] Sun H Lesche R Li DM Liliental J Zhang H Gao J PTEN modulates cell cycle progression and cell survival by regulating phosphatidylinositol 3,4,5,-trisphosphate and Akt/protein kinase B signaling pathway Proc Natl Acad Sci USA 1999 96 6199 6204 10339565 10.1073/pnas.96.11.6199 PMC26859

[bib14] Leslie NR Foti M Non-genomic loss of PTEN function in cancer: not in my genes Trends pharmacol sci 2011 32 131 140 21236500 10.1016/j.tips.2010.12.005

[bib15] Karreth FA Tay Y Perna D Ala U Tan SM Rust AG *In vivo* identification of tumor-suppressive PTEN ceRNAs in an oncogenic BRAF-induced mouse model of melanoma Cell 2011 147 382 395 22000016 10.1016/j.cell.2011.09.032 PMC3236086

[bib16] Tay Y Kats L Salmena L Weiss D Tan SM Ala U Coding-independent regulation of the tumor suppressor PTEN by competing endogenous mRNAs Cell 2011 147 344 357 22000013 10.1016/j.cell.2011.09.029 PMC3235920

[bib17] Jin G Kim MJ Jeon HS Choi JE Kim DS Lee EB PTEN mutations and relationship to EGFR, ERBB2, KRAS and TP53 mutations in non-small cell lung cancers Lung Cancer 2010 69 279 283 20018398 10.1016/j.lungcan.2009.11.012

[bib18] Soria JC Lee HY Lee JI Wang L Issa JP Kemp BL Lack of PTEN expression in non-small cell lung cancer could be related to promoter methylation Clin Cancer Res 2002 8 1178 1184 12006535

[bib19] Cai J Fang L Huang Y Li R Yuan J Yang Y miR-205 targets PTEN and PHLPP2 to augment AKT signaling and drive malignant phenotypes in non-small cell lung cancer Cancer Res 2013 73 5402 5415 23856247 10.1158/0008-5472.CAN-13-0297

[bib20] Sacco JJ Coulson JM Clague MJ Urbe S Emerging roles of deubiquitinases in cancer-associated pathways IUBMB Life 2010 62 140 157 20073038 10.1002/iub.300 PMC7165618

[bib21] Clague MJ Coulson JM Urbé S Cellular functions of the DUBs J Cell Sci 2012 125 277 286 22357969 10.1242/jcs.090985

[bib22] Clague MJ Barsukov I Coulson JM Liu H Rigden DJ Urbe S Deubiquitylases from genes to organism Physiol rev 2013 93 1289 1315 23899565 10.1152/physrev.00002.2013

[bib23] Nijman SM Luna-Vargas MP Velds A Brummelkamp TR Dirac AM Sixma TK A genomic and functional inventory of deubiquitinating enzymes Cell 2005 123 773 786 16325574 10.1016/j.cell.2005.11.007

[bib24] Reyes-Turcu FE Ventii KH Wilkinson KD Regulation and cellular roles of ubiquitin-specific deubiquitinating enzymes Annu Rev Biochem 2009 78 363 397 19489724 10.1146/annurev.biochem.78.082307.091526 PMC2734102

[bib25] Amodio N Scrima M Palaia L Salman AN Quintiero A Franco R Oncogenic role of the E3 ubiquitin ligase NEDD4-1, a PTEN negative regulator, in non-small-cell lung carcinomas Am J Pathol 2010 177 2622 2634 20889565 10.2353/ajpath.2010.091075 PMC2966817

[bib26] Maddika S Kavela S Rani N Palicharla VR Pokorny JL Sarkaria JN WWP2 is an E3 ubiquitin ligase for PTEN Nat cell biol 2011 13 728 733 21532586 10.1038/ncb2240 PMC3926303

[bib27] Van Themsche C Leblanc V Parent S Asselin E X-linked inhibitor of apoptosis protein (XIAP) regulates PTEN ubiquitination, content and compartmentalization J Biol Chem 2009 284 20462 20466 19473982 10.1074/jbc.C109.009522 PMC2742810

[bib28] Song MS Salmena L Carracedo A Egia A Lo-Coco F Teruya-Feldstein J The deubiquitinylation and localization of PTEN are regulated by a HAUSP-PML network Nature 2008 455 813 817 18716620 10.1038/nature07290 PMC3398484

[bib29] Omerovic J Clague MJ Prior IA Phosphatome profiling reveals PTPN2, PTPRJ and PTEN as potent negative regulators of PKB/Akt activation in Ras-mutated cancer cells Biochem J 2010 426 65 72 19922411 10.1042/BJ20091413 PMC3351670

[bib30] Rodriguez-Lebron E Costa MD Luna-Cancalon K Peron TM Fischer S Boudreau RL Silencing mutant ATXN3 expression resolves molecular phenotypes in SCA3 transgenic mice Mol ther 2013 21 1909 1918 23820820 10.1038/mt.2013.152 PMC3808130

[bib31] Vazquez F Ramaswamy S Nakamura N Sellers WR Phosphorylation of the PTEN tail regulates protein stability and function Mol Cell Biol 2000 20 5010 5018 10866658 10.1128/mcb.20.14.5010-5018.2000 PMC85951

[bib32] Wu X Hepner K Castelino-Prabhu S Do D Kaye MB Yuan XJ Evidence for regulation of the PTEN tumor suppressor by a membrane-localized multi-PDZ domain containing scaffold protein MAGI-2 Proc Natl Acad Sci USA 2000 97 4233 4238 10760291 10.1073/pnas.97.8.4233 PMC18208

[bib33] Mao JH Kim IJ Wu D Climent J Kang HC DelRosario R FBXW7 targets mTOR for degradation and cooperates with PTEN in tumor suppression Science 2008 321 1499 1502 18787170 10.1126/science.1162981 PMC2849753

[bib34] Yim EK Peng G Dai H Hu R Li K Lu Y Rak functions as a tumor suppressor by regulating PTEN protein stability and function Cancer Cell 2009 15 304 314 19345329 10.1016/j.ccr.2009.02.012 PMC2673492

[bib35] Yang Y Zhou F Fang Z Wang L Li Z Sun L Post-transcriptional and post-translational regulation of PTEN by transforming growth factor-beta1 J Cell Biochem 2009 106 1102 1112 19206163 10.1002/jcb.22100

[bib36] Song EJ Werner SL Neubauer J Stegmeier F Aspden J Rio D The Prp19 complex and the Usp4^Sart3^ deubiquitinating enzyme control reversible ubiquitination at the spliceosome Genes Dev 2010 24 1434 1447 20595234 10.1101/gad.1925010 PMC2895201

[bib37] Sowa ME Bennett EJ Gygi SP Harper JW Defining the human deubiquitinating enzyme interaction landscape Cell 2009 138 389 403 19615732 10.1016/j.cell.2009.04.042 PMC2716422

[bib38] Evert BO Araujo J Vieira-Saecker AM De Vos RA Harendza S Klockgether T Ataxin-3 represses transcription *via* chromatin binding, interaction with histone deacetylase 3 and histone deacetylation J Neurosci 2006 26 11474 11486 17079677 10.1523/JNEUROSCI.2053-06.2006 PMC6674535

[bib39] Li F Macfarlan T Pittman RN Chakravarti D Ataxin-3 is a histone-binding protein with two independent transcriptional corepressor activities J Biol Chem 2002 277 45004 45012 12297501 10.1074/jbc.M205259200

[bib40] Salmena L Poliseno L Tay Y Kats L Pandolfi PP A ceRNA hypothesis: the Rosetta stone of a hidden RNA language Cell 2011 146 353 358 21802130 10.1016/j.cell.2011.07.014 PMC3235919

[bib41] Poliseno L Salmena L Zhang J Carver B Haveman WJ Pandolfi PP A coding-independent function of gene and pseudogene mRNAs regulates tumor biology Nature 2010 465 1033 1038 20577206 10.1038/nature09144 PMC3206313

[bib42] Gan YH Zhang S PTEN/AKT pathway involved in histone deacetylases inhibitor induced cell growth inhibition and apoptosis of oral squamous cell carcinoma cells Oral Oncol 2009 45 e150 e154 19574087 10.1016/j.oraloncology.2009.05.563

[bib43] Pan L Lu J Wang X Han L Zhang Y Han S Histone deacetylase inhibitor trichostatin a potentiates doxorubicin-induced apoptosis by up-regulating PTEN expression Cancer 2007 109 1676 1688 17330857 10.1002/cncr.22585

[bib44] Marks PA Discovery and development of SAHA as an anticancer agent Oncogene 2007 26 1351 1356 17322921 10.1038/sj.onc.1210204

[bib45] Moss AC Jacobson GM Walker LE Blake NW Marshall E Coulson JM SCG3 transcript in peripheral blood is a prognostic biomarker for REST-deficient small cell lung cancer Clin Cancer Res 2009 15 274 283 19118055 10.1158/1078-0432.CCR-08-1163

[bib46] Seki T Gong L Williams AJ Sakai N Todi SV Paulson HL JosD1, a membrane-targeted deubiquitinating enzyme, is activated by ubiquitination and regulates membrane dynamics, cell motility and endocytosis J Biol Chem 2013 288 17145 17155 23625928 10.1074/jbc.M113.463406 PMC3682520

[bib47] Buus R Faronato M Hammond DE Urbe S Clague MJ Deubiquitinase activities required for hepatocyte growth factor-induced scattering of epithelial cells Curr Biol 2009 19 1463 1466 19699092 10.1016/j.cub.2009.07.040 PMC2764384

[bib48] Weeks SD Grasty KC Hernandez-Cuebas L Loll PJ Crystal structure of a Josephin-ubiquitin complex: evolutionary restraints on ataxin-3 deubiquitinating activity J Biol Chem 2011 286 4555 4565 21118805 10.1074/jbc.M110.177360 PMC3039388

[bib49] Winborn BJ Travis SM Todi SV Scaglione KM Xu P Williams AJ The deubiquitinating enzyme ataxin-3, a polyglutamine disease protein, edits Lys63 linkages in mixed linkage ubiquitin chains J Biol Chem 2008 283 26436 26443 18599482 10.1074/jbc.M803692200 PMC2546540

[bib50] Todi SV Winborn BJ Scaglione KM Blount JR Travis SM Paulson HL Ubiquitination directly enhances activity of the deubiquitinating enzyme ataxin-3 EMBO J 2009 28 372 382 19153604 10.1038/emboj.2008.289 PMC2646149

[bib51] Gorski JJ Pathak S Panov K Kasciukovic T Panova T Russell J A novel TBP-associated factor of SL1 functions in RNA polymerase I transcription EMBO J 2007 26 1560 1568 17318177 10.1038/sj.emboj.7601601 PMC1829371

[bib52] Vasudevan KM Gurumurthy S Rangnekar VM Suppression of PTEN expression by NF-kappa B prevents apoptosis Mol Cell Biol 2004 24 1007 1021 14729949 10.1128/MCB.24.3.1007-1021.2004 PMC321419

[bib53] Andersen CL Asmar F Klausen T Hasselbalch H Gronbaek K Somatic mutations of the CREBBP and EP300 genes affect response to histone deacetylase inhibition in malignant DLBCL clones Leukemia Res Reports 2013 2 1 3 10.1016/j.lrr.2012.10.002 PMC3850379 24371765

[bib54] Ikeda H Yamaguchi M Sugai S Aze Y Narumiya S Kakizuka A Expanded polyglutamine in the Machado-Joseph disease protein induces cell death *in vitro* and *in vivo* Nat Genet 1996 13 196 202 8640226 10.1038/ng0696-196

[bib55] Wang HL Hu SH Chou AH Wang SS Weng YH Yeh TH H1152 promotes the degradation of polyglutamine-expanded ataxin-3 or ataxin-7 independently of its ROCK-inhibiting effect and ameliorates mutant ataxin-3-induced neurodegeneration in the SCA3 transgenic mouse Neuropharmacology 2013 70 1 11 23347954 10.1016/j.neuropharm.2013.01.006

