# Supplementary material for: The deubiquitylase Ataxin-3 restricts PTEN transcription in lung cancer cells
Source: Oncogene. 2013 Dec 2;33(33):4265–72. doi: 10.1038/onc.2013.512 (PMC4351423; doi:10.1038/onc.2013.512)
Supplement: Supplementary Information [file onc2013512x1.doc]

**Supplementary Information**

Supplementary Table 1. RT-PCR primer pairs

Supplementary Table 2. siRNA sequences

**Supplementary Table 1. RT-PCR primer pairs**

| Gene | Forward Primer | Reverse Primer |
| --- | --- | --- |
| PTEN ^1^ | 5’-GTTTACCGGCAGCATCAAAT-3’ | 5’-CCCCCACTTTAGTGCACAGT-3’ |
| PTENP1 ^1^ | 5’-TCAGAACATGGCATACACCAA -3’ | 5’-TGATGACGTCCGATTTTTCA-3’ |
| ATXN3 | 5’-GTATGCAAGGTAGTTCCAGAAAC-3’ | 5’-TGTTGCTGCTTTTGCTGCTGT-3’ |
| JOSD1 | 5’-AAGATGCCCGAGTGGATTGG-3’ | 5’-TCCTCCAACTCTGATGAGCCTC-3’ |
| ATXN3L | 5’-GAACTAAGCCGCCAAGAAACC-3’ | 5’-GCTTCTGTTCCTGCTGATGC-3’ |
| SCG3 ^2^ | 5’-GATCCAGATGGTCTTCATCAAC-3’ | 5’-CTGATTCTCAGTCCAGCTTGTG-3’ |
| ACTB | 5’-CACCTTCTACAATGAGCTGCGTGTG-3’ | 5’-ATAGCACAGCCTGGATAGCAACGTAC-3’ |

^1^ Poliseno L, Salmena L, Zhang J, Carver B, Haveman WJ, Pandolfi PP (2010). A coding-independent function of gene and pseudogene mRNAs regulates tumour biology. Nature 465: 1033-1038.

^2^ Moss AC, Jacobson GM, Walker LE, Blake NW, Marshall E, Coulson JM (2009). SCG3 transcript in peripheral blood is a prognostic biomarker for REST-deficient small cell lung cancer. Clin Cancer Res 15: 274-283.

**Supplementary Table 2. siRNA sequences.**

| **Gene** | **siRNA name** | **Name in siRNA library** | **siRNA sequence** | **Supplier** |
| --- | --- | --- | --- | --- |
| ATXN3 | ATXN3_1 | ATXN3_D | 5’-TACGATGGGATCATTATTTCA-3’ | Qiagen |
| ATXN3 | ATXN3_3 | ATXN3_A | 5’-TCGGAAGAGACGAGAAGCCTA-3’ | Qiagen |
| ATXN3 | ATXN3_4 | ATXN3_D | 5’-TGCGTCGGTTGTAGGACTAAA-3’ | Qiagen |
| ATXN3 | ATXN3_5 | ATXN3_C | 5’-CAGGGCTATTCAGCTAAGTAT-3’ | Qiagen |
| PTEN | PTEN_6 | n/a | 5’-AAGGCGTATACAGGAACAATA-3’ | Qiagen |
| PTENP1 | PTENP1_2 | n/a | 5’-ATCAGAGATCATATAGGAATA-3’ | Qiagen |
| n/a | siC | n/a | All Stars Negative Control | Qiagen |
| n/a | NT1 | n/a | ON-TARGET Plus, non-targeting 1 | Dharmacon |
